# Notch2 Blockade Mitigates Methotrexate Chemotherapy-Induced Bone Loss and Marrow Adiposity

**DOI:** 10.3390/cells11091521

**Published:** 2022-05-02

**Authors:** Yaser Peymanfar, Yu-Wen Su, Cory J. Xian

**Affiliations:** UniSA Clinical and Health Science, City West Campus, University of South Australia, GPO Box 2471, North Terrace, Adelaide, SA 5001, Australia; yaser.peymanfar@mymail.unisa.edu.au (Y.P.); yu-wen.su@unisa.edu.au (Y.-W.S.)

**Keywords:** methotrexate, cancer chemotherapy, bone damage, bone recovery, Notch signalling, Wnt signalling

## Abstract

Childhood cancer methotrexate (MTX) chemotherapy often causes bone growth impairments, bone loss, and increased risks of fractures during or after treatment, for which the pathobiology is unclear and there is a lack of specific treatment. Our time course analyses of long bones from rats receiving intensive MTX treatment (mimicking a clinical protocol) found decreased trabecular bone volume, increased osteoclast formation and activity, increased adipogenesis in the expense of osteogenesis from the bone marrow stromal cells at days 6 and 9 following the first of five daily MTX doses. For exploring potential mechanisms, PCR array expression of 91 key factors regulating bone homeostasis was screened with the bone samples, which revealed MTX treatment-induced upregulation of Notch receptor *NOTCH2*, activation of which is known to be critical in skeletal development and bone homeostasis. Consistently, increased Notch2 activation in bones of MTX-treated rats was confirmed, accompanied by increased expression of Notch2 intracellular domain protein and Notch target genes *HEY1*, *HES1* and *HEYL*. To confirm the roles of Notch2 signalling, a neutralising anti-Notch2 antibody or a control IgG was administered to rats during MTX treatment. Microcomputed tomography analyses demonstrated that trabecular bone volume was preserved by MTX+anti-Notch2 antibody treatment. Anti-Notch2 antibody treatment ameliorated MTX treatment-induced increases in osteoclast density and *NFATc1* and *RANKL* expression, and attenuated MTX-induced bone marrow adiposity via regulating Wnt/β-catenin signalling and *PPARγ* expression. Thus, Notch2 signalling plays an important role in mediating MTX treatment-induced bone loss and bone marrow adiposity, and targeting Notch2 could be a potential therapeutic option.

## 1. Introduction

Methotrexate (MTX) is an anti-metabolite chemotherapeutic agent that affects cells through inhibition of dihydrofolate reductase resulting in a decreased supply of folates during de novo purine and pyrimidine synthesis, therefore disrupting DNA/RNA synthesis and cell replication [1]. MTX chemotherapy is mainly used for treatment of childhood cancers such as acute lymphoblastic leukemia (ALL) [2]. Additionally, it is used alone or in combination with other agents for treatment of osteosarcoma [3], breast cancer [4], and non-Hodgkin’s lymphoma [5]. However, with high rates of success in cancer chemotherapy, prevalence of skeletal complications such as growth impairments, decreased bone mineral density, increased risks of fracture, osteonecrosis, high bone marrow fat content (adiposity) and hematopoietic defects has been elevated in patients during and after chemotherapy [6,7,8,9,10,11]. Apart from these clinical investigations, rat model studies have also illustrated that intensive acute MTX treatment decreases trabecular bone formation, increases bone resorption and marrow fat accumulation in long bones [12,13,14,15,16]. Despite previous clinical and experimental reports on adverse effects of MTX on bone and bone marrow microenvironment, the mechanisms by which MTX disrupts bone homeostasis, and the bone/bone marrow can recover after chemotherapy are not fully understood. Additionally, with the lack of safe and specific therapeutics to prevent chemotherapy-induced bone loss, there is substantial need for safe and effective new therapeutics to be developed for protecting bone against chemotherapy side effects.

Trabecular bone osteoblasts (bone forming cells) and marrow adipocytes (fat cells) are derived from the common mesenchymal or stromal precursor cells (BMSCs) in the bone marrow. Previously, commitment of BMSCs to either fate as well as the associated regulatory mechanisms, particularly the Wnt/β-catenin signalling governing the bone/fat balance, have been studied extensively before under both physiological and pathological conditions [15,17]. MTX treatment has been shown to cause the bone/fat switch (increased adipogenesis in the expense of osteogenesis) [15,18], and attenuated Wnt signalling has been found as one of the potential mechanisms [19]. However, whether MTX alters Wnt signalling directly or indirectly by influencing changes in other regulatory pathways needs to be investigated. Moreover, previous studies have also demonstrated that MTX chemotherapy disrupts the homeostasis between bone resorption and bone formation by increasing the density and activity of osteoclasts (bone resorptive cells) in metaphyseal trabeculae in rats [16]. Although chemokines such as CXCL12 and pro-inflammatory cytokines such as TNF-α and receptor activator of nuclear factor kappa-Β ligand (RANKL) are known to have roles in controlling osteoclast formation and activity [20,21], and they have been shown to have altered expression in bones after MTX treatment [16,22], the molecular mechanisms underlying MTX-induced increased osteoclast formation and resorption are still not fully understood.

Notch is an evolutionarily conserved signalling that determines cell fates and functions and plays important roles in skeletal development and bone homeostasis [23]. In mammals there are four Notch receptors (Notch1-4) and five Delta/Serrate/Lag2 (DSL) ligands termed Jagged (Jag)1, Jag2, Delta-like (Dll)1, Dll3 and Dll4 [24]. Upon direct interaction of ligands and receptors, series of cleavage by metalloproteases release the Notch intracellular domain (NICD) that can then translocate to the nucleus and induce transcription of Notch target genes including hairy and enhancer of split (Hes) and Hes-related with YRPW motif (Hey) [25,26]. The impact of Notch signalling, particularly that of Notch2 on skeleton homeostasis, has been well studied in numerous skeletal disorders and abnormalities such as Hajdu–Cheney syndrome (HCS) [23,27]. Initial in vitro studies revealed that overactivation of Notch receptors suppress osteoblastic differentiation [28,29]. Conditional deletion of *Notch2* in BMSCs increased trabecular bone formation in mice [30]. Additionally, systemic *Notch2* gain of function in growing mice caused femoral growth arrest, indicating a principal role of Notch (particularly Notch2) pathway in modulation of bone growth [31]. Furthermore, enhanced bone resorption and reduced osteoblastic function has been shown in *Notch2* gain of function mutants [31,32]. Supporting this, one study using mesenchymal cell-specific Notch2^fl/fl^/Prx-Cre knockout mice has observed a higher bone volume, although this phenotype has not been observed in Notch1^fl/fl^/Prx-Cre mice, confirming the stronger function of Notch2 over Notch1 in controlling bone formation [33].

Several studies indicated the crosstalk between Notch signalling and Wnt pathway in the skeleton at different stages and revealed that Notch and Wnt pathways have mostly opposite roles in bone homeostasis [34,35,36]. Notch activation suppressed β-catenin in osteoblast lineage cells and reduced osteoblast differentiation [37,38]. Notch signalling was shown to have key roles in increasing BMSC proliferation and commitment towards adipocytes [17,39]. Furthermore, Notch signalling controls osteoclastogenesis by regulating expression of osteoprotegerin (OPG) and RANKL in osteoblasts [32,40]. Additionally, it has been illustrated that Notch2 induces osteoclastogenesis via enhancing expression of nuclear factor of activated T-cells 1 (NFATc1) [41]. Recently, in an in vitro study, we have found that MTX treatment-suppressed osteoblastic differentiation was associated with induced Notch2 signalling and Notch2 blockade attenuated MTX adverse effect in osteogenesis by activating Wnt/β-catenin signalling [42]. Despite previous studies on roles of Notch2 in skeletal development and diseases, there is a lack of knowledge about the potential roles of Notch2 signalling in cancer chemotherapy-induced bone defects.

In the current study, a PCR array of 91 selected genes with known roles in bone homeostasis was screened with bone samples of rats treated with MTX, which revealed altered expression of some components of Notch signalling pathway. In addition, suggesting important roles of the Notch2 signalling in mediating MTX treatment-induced bone and bone marrow defects in rats, administration with a neutralising antibody against the negative regulatory region of Notch2 was found to ameliorate MTX-induced bone damage and bone marrow adiposity as well as the change in Wnt/β-catenin signalling.

## 2. Materials and Methods

### 2.1. Rat MTX Chemotherapy Time-Course Study

Six-week-old male Sprague Dawley rats (120–150 g approximately) received 5 consecutive doses of MTX at 0.75 mg/kg/day subcutaneously as described [13,43]. A group of saline-injected rats was used as a normal control group. Bone specimens were collected for histological, cellular, gene expression and protein level analyses on days 6, 9 and 14 following the initial dose (n = 5 rats/group). The protocol, including major regulatory aspects for rodent experimentation procedures, was approved by the Animal Ethics Committee of the University of South Australia.

### 2.2. PCR Array Analysis

To investigate MTX treatment effects on expression of 91 key regulatory factors governing bone homeostasis (Supplementary Table S1), a quantitative mRNA expression analysis was performed using a rat custom RT^2^ profiler PCR array kit (CLAR31177; Qiagen, Germantown, MD, USA) with RNA samples extracted (see below) from rats at day 9 after the first MTX dose. Quantitative PCR was conducted as described below, and relative gene expression levels were calculated using the 2^−ΔΔCt^ method. House-keeping genes ribosomal protein P1 and lactate dehydrogenase A were used as internal controls.

### 2.3. Anti-Notch2 Treatment Study

Following the PCR array gene expression analyses, Notch2 was chosen as a target for the subsequent intervention study. Saline- or MTX-treated rats were randomly divided into four groups receiving control IgG or anti-Notch2-neutralising IgG, Saline + control IgG, Saline + anti-Notch2 antibody, MTX + control IgG, and MTX + anti-Notch2, respectively (n = 10 rats/group, n = 6 for histological, cellular, and molecular analyses and n = 4 for micro-computed tomography (micro-CT) analyses (SkyScan 1276, Bruker, Kontich, Belgium)). Anti-ragweed control IgG and anti-Notch2 IgG against the negative regulatory region of Notch2 (NRR2) (kindly supplied by Genentech, South San Fransisco, CA, USA) were suspended in phosphate-buffered saline (PBS) and administrated intraperitoneally at a dose of 5 mg/kg (a suggested effective dose without causing gastrointestinal toxicity [44]) every 3 days (day 1, 4 and 7 following first MTX injection). The dose of antibody was chosen based on previous in vivo studies [44,45,46]. Specimens were collected at day 9, a time point that was previously found to have most apparent histological damages in bone and bone marrow following MTX treatment [13]. The protocol, including major regulatory aspects for rodent experimentation procedures, was approved by the Animal Ethics Committee of the University of South Australia. Following euthanasia by CO_2_ overdose, tibiae, femurs, and pelvises were dissected. The left tibia, including proximal epiphysis, growth plate and adjacent metaphyseal and diaphyseal bone (about 1 cm), were collected, fixed in 10% neutral-buffered formalin for 24 h and decalcified in Immunocal (Decal Corp, Tallman, New York, NY, USA) for 21 days at 4 °C, processed and embedded in paraffin wax [19]. The metaphysis was collected from the right tibia and was snap frozen in liquid nitrogen and stored at −80 °C for RNA isolation [15]. To collect bone marrow cells for cell culture studies, dissected femurs, pelvises and humeri of each animal were placed in Eppendorf tubes and centrifuged at 900 rcf prior to the cell portion being pulled together. For micro-CT analyses, tibias were collected and kept in 80% ethanol until scanning.

### 2.4. Micro-Computed Tomography (Micro-CT)

Microarchitecture of tibias from both treatments and control rats (n = 4) was determined by micro-CT scanning using Skyscan 1276 (Bruker, Kontich, Belgium) equipped with an X-ray source working at 55 kV/72 µA. All tibial bones were scanned in tubes with 80% ethanol, fixed on scanning bed at a pixel size of 6.5 µm, rotation step set at 0.4° and exposure performed with 0.25 mm aluminium filter. Reconstruction of scanned images was performed using NRecon, analysed by CTAn software (Skyscan) and 3D images were constructed using CTvol (Skyscan). The region for analysis was selected from 0 to 2 mm below the growth plate from the transverse plane images in a total of 155 layers (consisting mainly of primary and secondary spongiosa). Trabecular regions were quantified for trabecular bone volume/total volume ratio (BV/TV%), trabecular thickness (mm), number (per mm) and spacing (mm) using adaptive (mean of min and max values) thresholds [18].

### 2.5. Histological Cell Density Analyses

To examine treatment effects on cell densities, sections of 4 µm from paraffin-embedded tibial specimens were stained by H&E and analysed. Osteoblasts were recognized by their cuboidal shape and their location (lining trabecular or endosteal bone surfaces) and counted as cells/mm^2^ trabecular area at the secondary spongiosa region [19]. Adipocytes were enumerated per millimetre square of bone marrow area at the lowest region of secondary spongiosa, as recognized by their typical shape, size, and location. To examine treatment effects on osteoclast number, bone sections were stained for tartrate resistant acid phosphatase (TRAP) and counterstained with hematoxylin as described [47]. TRAP^+^ osteoclasts on bone trabeculae of secondary spongiosa were counted and expressed as osteoclast number/mm^2^ [19].

### 2.6. RNA Isolation and Quantitative RT-PCR Analyses of Gene Expression

To examine treatment effects on gene expression of selected osteogenic and adipogenic markers, and some components of Notch signalling and Wnt/β-catenin pathways, real-time quantitative RT-PCR analyses were conducted with RNA isolated from frozen metaphyseal bone tissue as described [14,15]. Equal amounts of RNA were reverse transcribed using the iScript Select cDNA synthesis kit (Bio-Rad, Hercules, CA, USA). Quantitative RT-PCR assays were performed on a CFX Connect PCR System (Bio-Rad) using Sso advanced universal SYBR Green super mix kit (Bio-Rad) with specific primers (Table 1, designed with PRIMER-BLAST, and supplied by Sigma-Aldrich, NSW, Australia for Notch signalling and by Gene-Works, Adelaide, Australia for other primers). Relative expression was calculated using quantitative Ct (2^−ΔCt^) method against Cyclophilin A as the endogenous control.

### 2.7. Immunohistochemistry

To assess treatment effect on the level of Notch2 receptor protein expression, immunohistochemistry was conducted on bone sections collected from the MTX time course study. Briefly, sections were deparaffinized, gradually hydrated and were quenched in 3% H_2_O_2_ and incubated in Tris EDTA (pH 9) for antigen retrieval. After blocking with 5% Pig serum, 4%BSA, 0.1% Triton-X 100, 0.05% Tween 20 for 60 min, sections were then incubated with primary antibody rabbit anti-Notch2-cleaved N-terminus (Merck Millipore, Darmstadt, Germany) (1:100 in a dilution buffer with 2% BSA and 0.25% Triton-X 100) overnight at 4 °C in a humidified chamber. Reaction was detected with biotinylated secondary antibodies swine anti-rabbit IgG (1:300) (Dako, North Sydney, Australia) for 60 min, and streptavidin-HRP (1:700) (R & D systems, Minneapolis, MN, USA) for 60 min at room temperature. Sections were developed with DAB Plus chromogen (Dako) and counterstained with Hematoxylin [48]. Replacement of the primary antibody with 1% bovine serum albumin in phosphate-buffered saline (PBS) or normal rabbit IgG at the same concentration was used following the same protocol as a negative control. Images were taken and analysed as previously described [48].

### 2.8. Ex Vivo CFU-f/ALP Assays

To investigate the treatment effect on osteogenic commitment of BMSCs and their potential to differentiate to osteoblasts, bone marrow mononuclear cells (BMMNC) from each rat separately were isolated by lymphoprep^TM^ (Axis-Shield, Oslo, Norway). The BMMNC pellet from each rat was resuspend in basal medium, containing α-MEM supplemented with 10% foetal bovine serum, 1% L-glutamine, 50 µg/mL Pen/Strep mix, 15 mM HEPES and 130 µM L-ascorbate-2-phosphate and cells were cultured in duplicate per rat at the density of 1 × 10^6^ cells/well in six-well plates. adhered BMSCs were further cultured for 14 days. Cells were fixed with 10% neutral-buffered formalin and stained with alkaline phosphatase (ALP) followed by staining with toluidine blue (Sigma-Aldrich, Sydney, NSW, Australia). ALP positive colonies and total (toluidine blue positive) were counted and expressed as %ALP^+^ of total colonies per well [22]. 

### 2.9. Ex Vivo Osteogenesis and Mineralisation Assays

In order to assess treatment effects on osteogenesis and mineralisation potential of isolated stromal cells, BMSCs isolated as described above were plated in duplicate for each rat at the density of 4 × 10^6^ cells/well in six-well plates and cultured with basal medium for 7 days and then for an additional 11 days in osteogenic induction medium consisting of basal medium supplemented with 10 nM dexamethasone and 10 mM β-glycerolphosphate, with the media being changed twice a week [19]. At day 11 of osteogenic induction, cells were formalin fixed and mineralisation potential was assessed by Alizarin red staining and colorimetric detection at 405 nm as described [49]. 

### 2.10. Ex Vivo Adipogenesis Assays

To examine the influence of treatment on adipogenesis potential of isolated BMSCs, BMSCs were cultured at 4 × 10^6^ cells/well in six-well plates in basal medium for 7 days and then a further 7 days in adipogenic induction medium containing basal medium supplemented with 1 µM dexamethasone (Sigma-Aldrich, Sydney, NSW, Australia), 0.5 mM methyl-isobutylxanthanine (IBMX) and 100 µM indomethacin (Sigma-Aldrich). Cells were stained with Nile red and DAPI (2-(4-amidinophenyl)-1H-indole-6-carboxamidine) and imaged by Zoe fluorescent cell imager (Bio-Rad) [19]. Adipogenic potential of BMSCs was expressed as % Nile red-positive cells over total cells counted.

### 2.11. Ex Vivo Osteoclastogenesis Assays

To investigate impact of treatment on osteoclastogenic potential in bone marrow, collected non-adherent cells from BMMNC culture obtained from each animal were plated at 3 × 10^5^ cells/well in a 96-well plate and cultured in basal medium supplemented with 10 ng/mL M-CSF (PeproTech, London, UK) overnight. On the following day, medium was replaced with basal medium containing 10 ng/mL M-CSF and 30 ng/mL RANKL (PeproTech). The culture was maintained for 7 days and then formalin fixed. Osteoclasts were identified by TRAP staining and stained cells with three or more nuclei were considered as osteoclasts and counted (cells/mm^2^) [16]. 

### 2.12. Western Blot

To examine treatment effects on levels of activated Notch2 (Notch2-cleaved N-terminus) and total cytosolic β-catenin levels, total tissue protein was isolated from tibial metaphysis collected from control and treated rats as described [50]. Protein concentrations were determined by the bicinchoninic acid (BCA) protein assay kit (Thermo Fisher, Melbourne, VIC, Australia). Total protein of 10 µg was run on a 4–20% pre-cast polyacrylamide TGX mini gel (Biorad) and transferred onto a Trans-Blot turbo Nitrocellulose membrane (Biorad) using Trans-Blot turbo transfer system (Biorad). A standard total protein staining method was used as an internal loading control (Li-Cor Bioscience, Lincoln, NE, USA) and images for total protein were taken with near infra-red Odyssey CLx imaging system (Li-Cor Bioscience). After blocking with 5% skim milk/TBST, membranes were probed with primary antibody for β-catenin (9562S) (1:1000) (Cell Signalling Technology, Danvers, MA, USA) and primary antibody for cleaved NICD2 (1:1000) (Merck Millipore, Darmstadt, Germany) in blocking buffer and incubated at 4 °C overnight, followed by 1 h room temperature incubation with a secondary antibody IRDye 800 CW donkey anti-rabbit (Li-Cor Bioscience), and then imaged with Odyssey CLx imaging system.

### 2.13. Statistics Analyses

Data was expressed as mean ± SEM and analysed with standard one-way ANOVA with a Tukey’s multiple comparison test using GraphPad Prism (8.3.0 for Windows, GraphPad Software, San Diego, CA, USA). Significance was considered when *p* < 0.05. In the graphs, asterisks on bars are representative of significant differences between the indicated columns compared to the control.

## 3. Results

### 3.1. MTX Chemotherapy Altered mRNA Expression of Key Regulatory Factors for Bone Homeostasis

**Figure 1:**
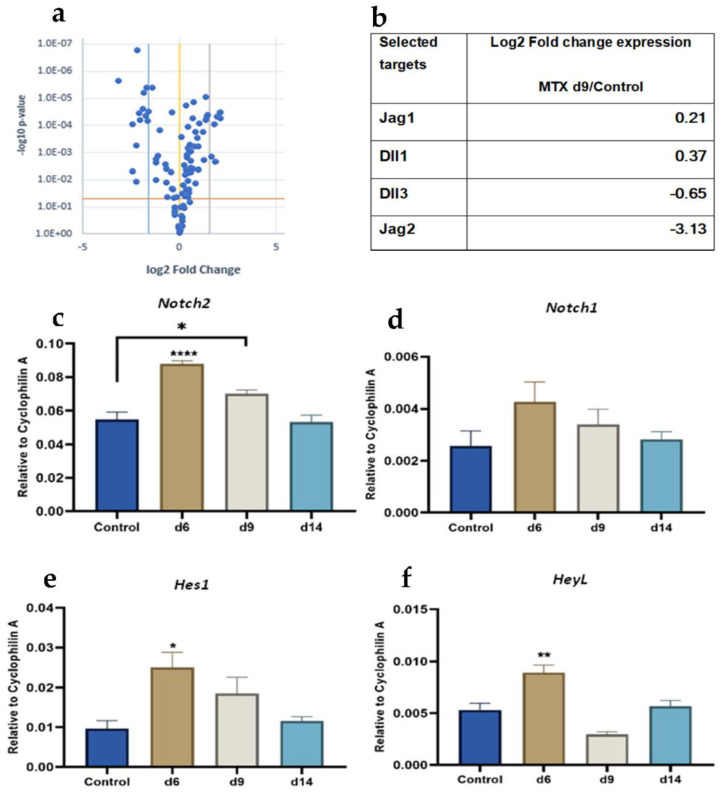
MTX treatment effects on mRNA expression of 92 key factors known important in regulating bone homeostasis and important Notch mediators over the MTX damage/recovery time-course vs. control. (**a**) the volcano plot represents logarithmic fold changes in mRNA expression of 92 factors in MTX day 9 vs. control rats. The yellow line indicates fold changes (2^−ΔΔCt^) in gene expression of 1 and the blue and grey colour lines indicate the desired fold-change in gene expression threshold defined as 3 as standard. The orange line indicates the desired threshold for the p value of the t-test defined as 0.05. (**b**) The most significant changes in mRNA expression have been observed in Notch ligand Jag2, which has remarkable logarithmic fold downregulation at day 9 following MTX treatment compared to control group. Quantitative RT-PCR relative gene expression analysis of (**c**) Notch2, (**d**) Notch1, (**e**) Hes1, (**f**) HeyL, using RNA isolated from metaphyseal bone specimens from control and MTX-treated rats; * *p* < 0.05, ** *p* < 0.001, **** *p* < 0.0001.

Based on our recent findings on cellular and pathological changes following MTX chemotherapy [2,13,19,51,52] and to investigate potential mechanisms for the bone damage, a PCR array was designed and used for detecting changes in mRNA expression levels of 91 key factors of major signalling pathways (known important in regulating bone homeostasis) (Supplementary Table S1) in bone samples collected from control rats or rats day 9 after the first of the five daily MTX dose at 0.75 mg/kg. As shown in the volcano plot analyses for the gene expression of the 91 factors (Figure 1a,b), MTX treatment significantly downregulated some of the Notch pathway ligands (e.g., Jag2, and Dll3) at day 9 compared to untreated controls, while there was one-fold upregulation for other Notch ligands (Dll1 and Jag1).

### 3.2. Time Course Analyses of MTX Treatment Effects on mRNA Expression Levels of Notch Receptors and Target Genes

Notch signalling has been linked to maintaining homeostasis of bone (by regulating bone formation, resorption, and marrow adipogenesis). Given the results obtained from our PCR array and based on previous studies on roles of Notch signalling particularly that of Notch2 and Notch1 receptors in regulating bone formation and resorption [23,32,33,46,53] as well as its known crucial role in controlling osteogenic/adipogenic commitment of BMSCs [17], the current study examined mRNA expression of most studied Notch signalling mediators (Notch2, Notch1 and Notch target genes) in metaphyseal bone samples of rats at different time points following MTX treatment (Figure 1c–f). As examined by quantitative RT-PCR, when compared to controls, while there were no significant changes in *Notch1* levels (Figure 1d), there was a remarkable upregulation of *Notch2* mRNA at day 6 (*p* < 0.0001) and day 9 (*p* < 0.05) following initial MTX dose, before it returned to the control level at day 14, a time point previously found to have bone recovery histologically (Figure 1c). Consistently, Notch target genes were found to be upregulated most significantly on day 6 (*Hes1*, *p* < 0.05; and *HeyL*, *p* < 0.01) (Figure 1e,f). These results suggest that the Notch2 signalling pathway may play a role in MTX chemotherapy-induced bone damage and subsequent recovery.

### 3.3. MTX Treatment Induced NICD2 Protein Expression Predominantly in Osteoblasts and Osteocytes in Metaphysis

**Figure 2:**
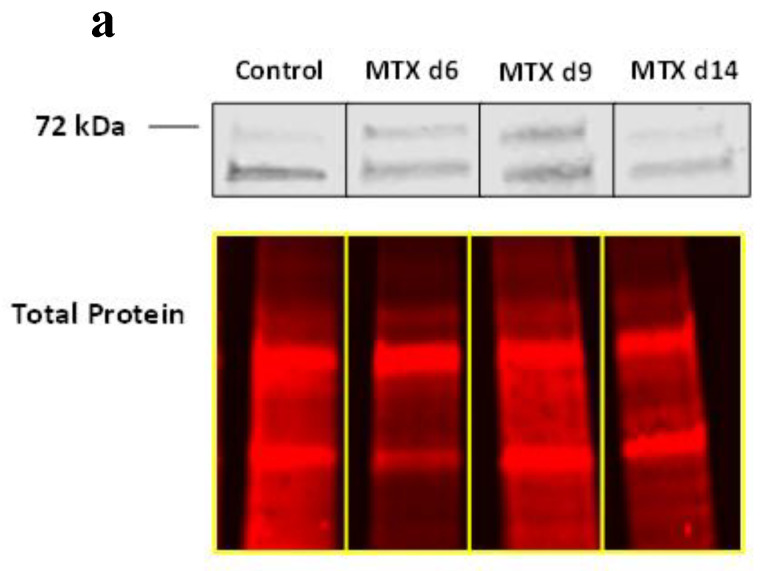

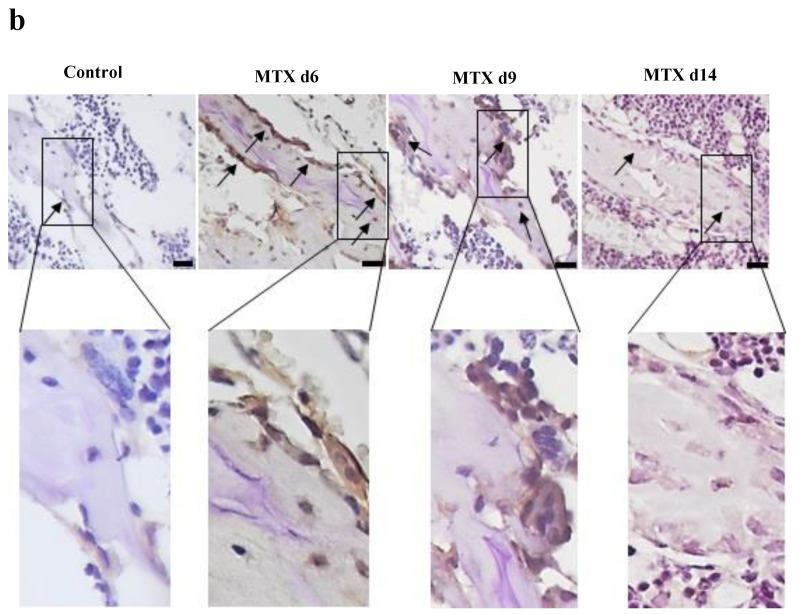
MTX treatment increases NICD2 protein levels following MTX treatment as assessed by Western blot and immunohistochemistry staining. (**a**) Metaphyseal bone NICD2 protein expression at different time points as assessed by Western blot with total protein being used as an internal loading control; (**b**) Immunohistochemistry of bone samples at different time points, with day 6 and day 9 MTX samples illustrating strong positivity for NICD2 protein in lining osteoblasts and osteocytes as indicated by black arrows, and control and day 14 MTX bone samples having faint staining of NICD2. Images were taken from lower region of the metaphysis (scale bar is 20 µm).

When protein expression of activated Notch2 (Notch2 cleaved N-terminus) was further assessed by Western blotting using metaphyseal bone samples, a trend of increased NICD2 expression at days 6 and 9 was seen (Figure 2a and Supplementary Figure S1). Further, to validate this change in protein expression and to determine prominent cells expressing NICD2, its immunohistochemical (IHC) staining was performed using bone sections from the MTX treatment time course. Consistent with Western blot results, IHC results confirmed obvious elevation of NICD2 at days 6 and 9 compared to controls, which was expressed most predominantly in bone lining osteoblasts and osteocytes in rat metaphyseal bone (Figure 2b).

### 3.4. Notch2 Blockade Alleviated MTX Adverse Effects in Metaphysis Bone Volume and Structure

**Figure 3:**
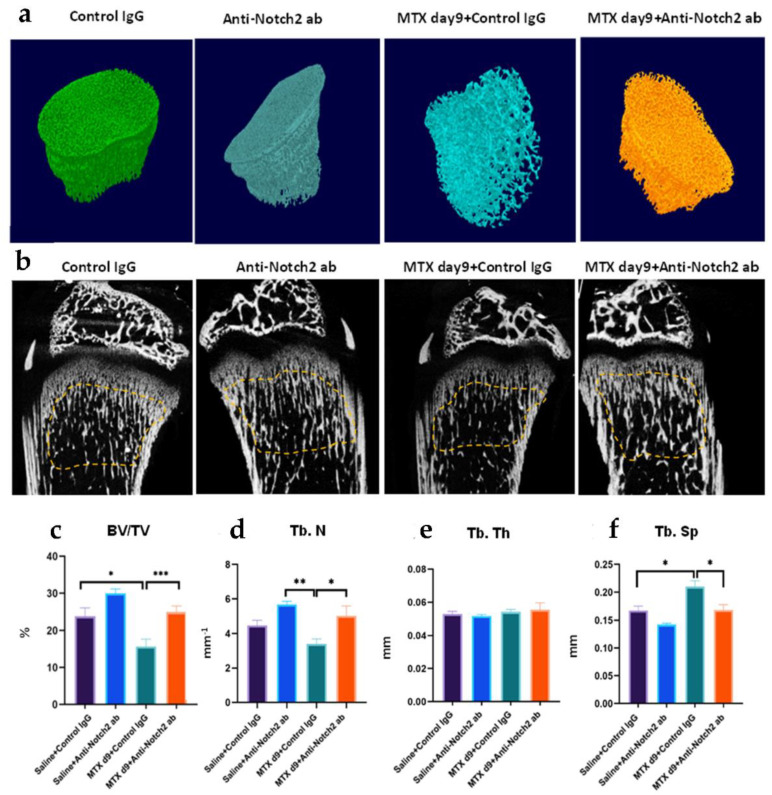
Effect of MTX or MTX + anti-Notch2 treatment on metaphysis volume and micro-architecture at day 9 after the first of 5 daily MTX treatments. (**a**) Micro-CT 3D images showing treatment effects on different groups; (**b**) micro-CT longitudinal cross-sections of tibia, with dotted lines marking the area of bone being analysed; (**c**) treatment effects on bone volume/tissue volume fraction (BV/TV%); treatment effects on (**d**) trabeculae number, (**e**) trabecular thickness, and (**f**) trabecular separation; * *p* < 0.05, ** *p* < 0.01, *** *p* < 0.001.

Next, to confirm the roles of Notch2 signalling in MTX bone damage, a neutralising anti-Notch2 antibody or a control IgG was administered to rats during MTX treatment. Micro-CT morphological analyses of tibial metaphysis trabecular bone volume and structure revealed obvious changes in the trabecular bone following MTX treatment (Figure 3a,b). While there was a significant reduction in ratio of trabecular bone volume per tissue volume (BV/TV%) following MTX + control IgG treatment (*p* < 0.05 vs. control), this reduction was remarkably attenuated in MTX + anti-Notch2 treatment group (*p* < 0.001 vs. MTX+control IgG) (Figure 3c). In addition, while MTX treatment showed a trend of reducing the trabecular number when compared to the normal control, blocking Notch2 along with MTX treatment remarkably attenuated the trabecular number reduction (*p* < 0.05 vs. MTX + control IgG) (Figure 3d). Furthermore, while trabecular thickness remained unchanged among all groups (Figure 3e), trabecular separation was increased following MTX treatment (*p* < 0.05 vs. controls), which was alleviated following MTX + anti-Notch2 treatment (*p* < 0.05 vs. MTX + control IgG) (Figure 3f). Consistent with micro-CT analyses, histomorphometry assessment on H&E-stained bone sections obtained similar results on the treatment effects on metaphysis trabecular bone volume and structure (data not shown).

### 3.5. Notch2 Blockade Attenuated MTX Treatment-Induced Increases in Osteoclast Formation and Expression of Osteoclastogenesis-Related Genes

**Figure 4:**
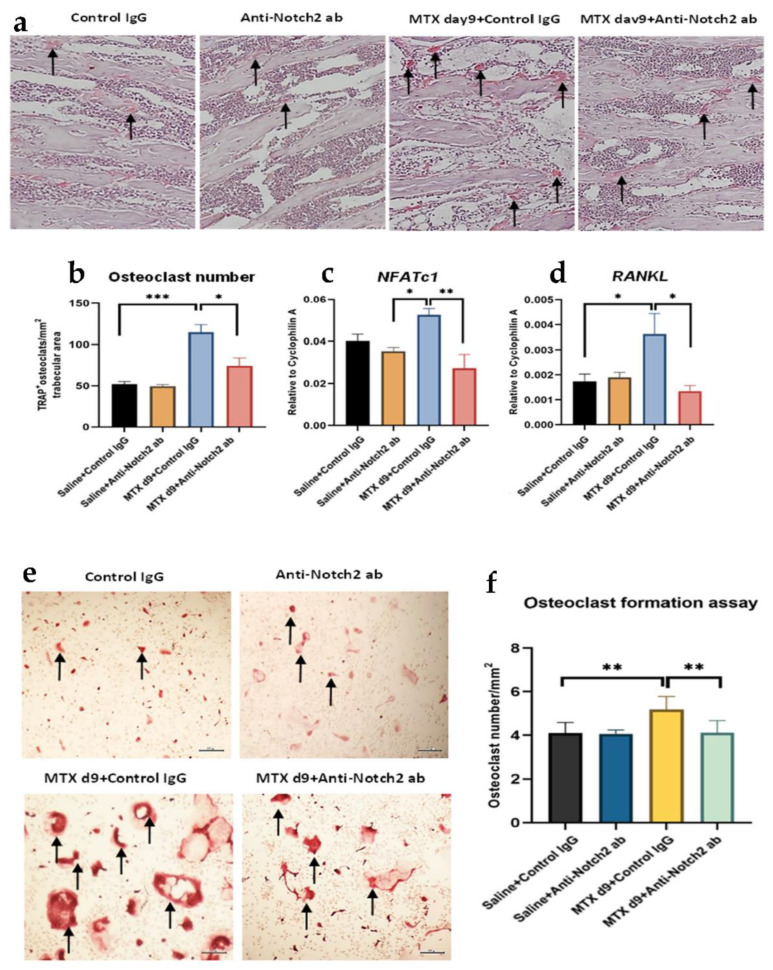
Treatment effects on osteoclast density, expression of regulatory genes and osteoclastogenic potential of non-adherent bone marrow cells isolated from rat bones. (**a**) Representative bone section images of different treatment groups with arrows pointing TRAP-stained multinucleated osteoclasts in the secondary spongiosa area; (**b**) osteoclast density at different treatment groups; treatment effects on (**c**) NFATc1 and (**d**) RANKL mRNA expression. (**e**) Representative images of osteoclast formation assays conducted for different group of rats, with TRAP-stained multinuclear osteoclasts being indicated by arrows; scale bar is 200 µm. (**f**) The graph indicates osteoclast formation potentials of different groups expressed as number of TRAP+ osteoclasts/mm^2^ culture area; * *p* < 0.05, ** *p* < 0.01, *** *p* < 0.001.

To determine whether the alterations observed in metaphyseal bone volume and structure were associated with a change in bone resorption, bone-resorbing tartrate-resistant acidic phosphatase positive (TRAP^+^) osteoclasts in secondary spongiosa area of metaphysis bone were counted histologically, and mRNA expression of key genes regulating osteoclast formation and activity were analysed. MTX treatment alone drastically increased the osteoclast density (*p* < 0.001 vs. control), which was significantly diminished by co-treatment with anti-Notch2 antibody (*p* < 0.05 vs. MTX) (Figure 4a,b). Quantitative RT-PCR analyses revealed that MTX treatment upregulated *NFATc1* mRNA expression, which was attenuated by the MTX + anti-Notch2 antibody treatment (*p* < 0.01 vs. MTX) (Figure 4c). Similarly, while MTX + control IgG induced RANKL expression (*p* < 0.05 vs. control), blockade of Notch2 along with MTX treatment alleviated this induction (*p* < 0.05 vs. MTX) (Figure 4d). Consistently, analyses of osteoclastogenic potential of non-adherent bone marrow cells isolated from treated rats illustrated that MTX treatment-induced increase in formation of TRAP^+^ multinucleated osteoclasts (*p* < 0.01 vs. control) was significantly attenuated by the MTX + anti-Notch2 antibody co-treatment (*p* < 0.01 vs. MTX + control IgG) (Figure 4e,f).

### 3.6. Notch2 Blockade Attenuated MTX Treatment-Induced Reciprocal Changes in Marrow Fat Volume

**Figure 5:**
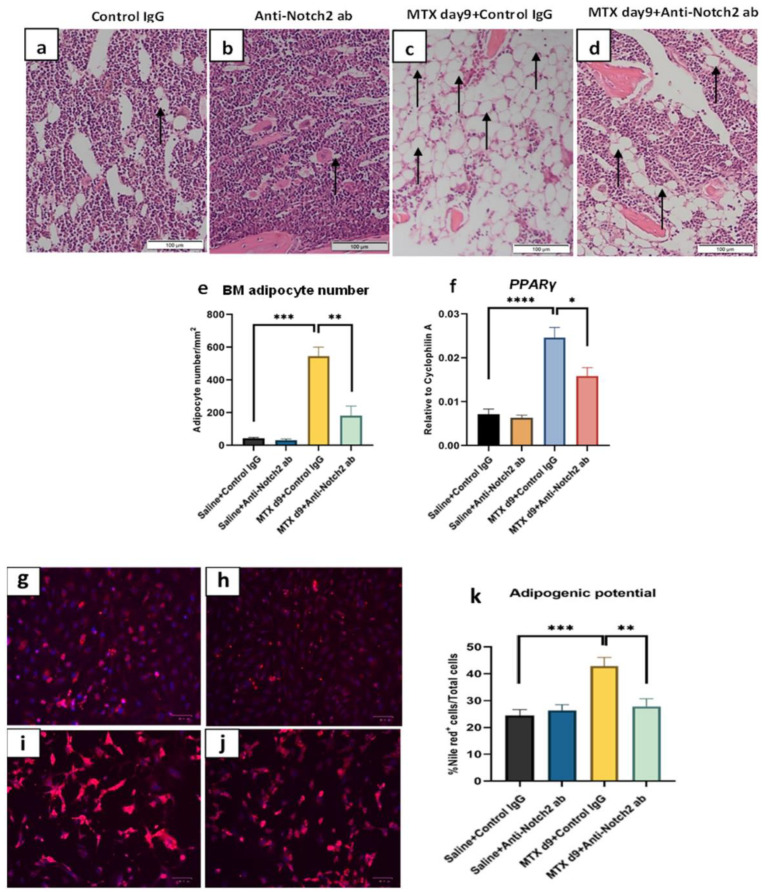
Notch2-neutralising antibody treatment rescued MTX-induced marrow adiposity. Histological images of H&E-stained tibial bone sections (showing mainly bone marrow) of different groups: (**a**) Control IgG, (**b**) anti-Notch2 antibody alone treated, (**c**) MTX + Control IgG treated, and (**d**) MTX + Anti-Notch2 treated; arrows pointing adipocytes; scale bar is 100 µm. (**e**) Treatment effects on bone marrow fat cell density, and (**f**) changes in mRNA expression of adipogenic transcription factor PPARγ. Representative images for adipogenic cultures with Nile red staining of different groups: (**g**) control IgG group, (**h**) anti-Notch2 antibody control, (**i**) MTX + Control IgG, (**j**) MTX + anti-Notch2 antibody group. Red colours represent stained intracellular lipid droplets and blue colours dapi stained nuclei; scale bar is 100 µm. (**k**) Quantification of adipogenic potential assessed by Nile red staining and expressed as percentage of Nile red + cells; * *p* < 0.05, ** *p* < 0.01, *** *p* < 0.001, **** *p* < 0.0001.

Histological assessment of H&E-stained tibial sections revealed a significant increase in the number of bone marrow adipocytes (fat cells) in the MTX + control IgG group (*p* < 0.001 vs. control) (Figure 5a–e). However, MTX treatment along with Notch2 blockade significantly attenuated MTX-induced marrow adiposity (*p* < 0.01) (Figure 5d,e). Consistently, quantitative RT-PCR gene expression analyses also showed that, upregulation of key adipogenic transcription factor PPARγ following MTX + control IgG (*p* < 0.0001 vs. control) was ameliorated with MTX + anti-Notch2 antibody co-treatment (*p* < 0.05 vs. MTX + control IgG) (Figure 5f).

### 3.7. Notch2 Blockade Ameliorated MTX Treatment-Induced Increase in Adipogenesis and Reduced Osteogenesis in Isolated BMSCs

**Figure 6:**
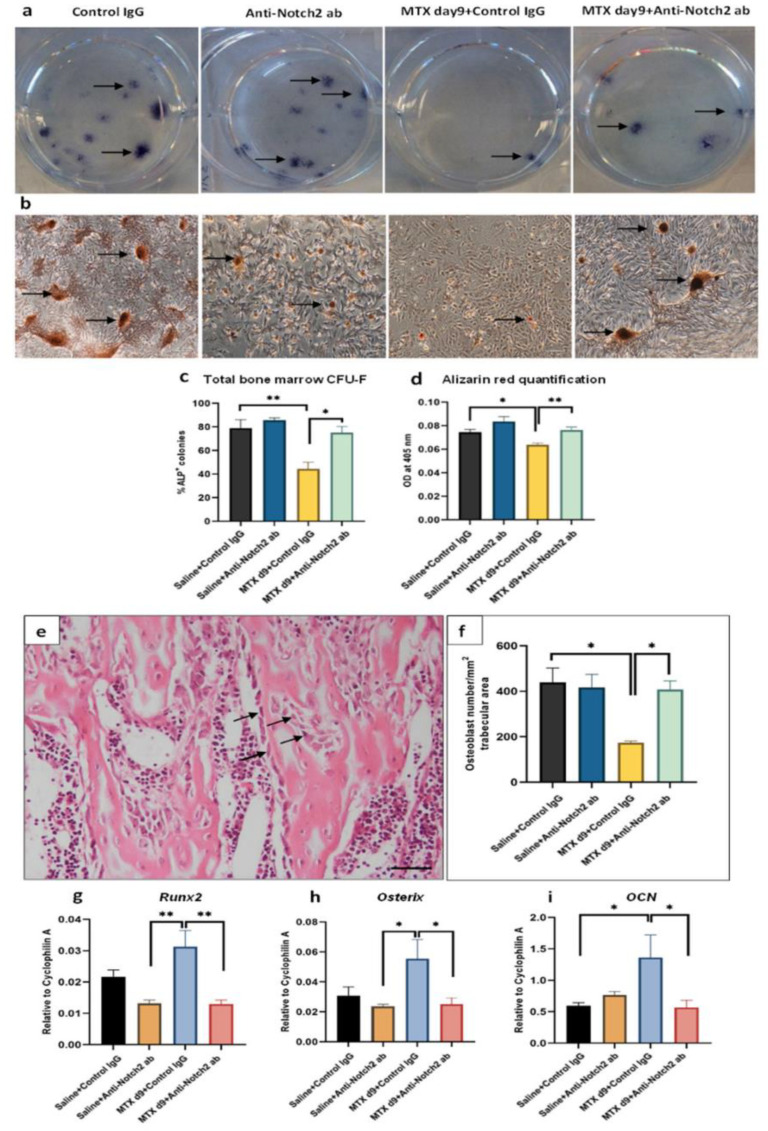
Treatment effects on osteogenic potential of bone marrow stromal cells (BMSCs), osteoblast density at secondary spongiosa and mRNA expression of osteogenesis regulatory genes in the metaphyseal bone. (**a**) Representative CFU-f colonies (arrows) stained positive for alkaline phosphatase (ALP) with BMSCs from different treatment groups; (**b**) representative images of alizarin red-stained mineralised nodules (arrows); scale bar is 200 µm. (**c**) Treatment effects on numbers of ALP+ CFU-f colonies; (**d**) alizarin red quantification with absorbance reading at 405 nm. (**e**) Location and morphology of osteoblasts in secondary spongiosa. Arrows highlight cuboidal osteoblasts. Scale bar 50 µM; (**f**) osteoblast density in tibial secondary spongiosa; treatment effects on mRNA expression in metaphyseal bone of (**g**) Runx2, (**h**) SP7 (Osterix), and (**i**) BGLAP (osteocalcin or OCN); * *p* < 0.05, ** *p* < 0.01.

Consistent with histological changes in bone marrow adiposity, ex vivo adipogenesis assays with BMSCs isolated from treated rats revealed that adipogenic potential of BMSCs was elevated significantly by MTX + control IgG treatment (*p* < 0.001 vs. control), which was remarkably rescued in the MTX + anti-Notch2 co-treatment (*p* < 0.01 vs. MTX + control IgG) (Figure 5g–k). On the other hand, assessments of the osteogenic potential of isolated BMSCs by ex vivo CFU-f assays revealed a significantly lower percentage of ALP^+^ colonies formed by BMSCs from the MTX + control IgG treated rats (*p* < 0.01 vs. control) (Figure 6a,c), and this reduction was significantly compensated with MTX + anti-Notch2 antibody co-treatment (*p* < 0.05) (Figure 6a,c). Similarly, the mineralisation potential of BMSCs (as assessed by mineralised nodules formed and quantified by Alizarin red staining from the ex vivo mineralisation assays) was reduced in MTX + control IgG group (*p* < 0.05 vs. control), but obviously improved with MTX + anti-Notch2 antibody co-treatment (*p* < 0.01 vs. MTX + control IgG) (Figure 6b,d).

### 3.8. Treatment Effects on Osteoblast Density and Gene Expression of Key Regulatory Osteogenic Factors

Density and activity of osteoblasts directly influence bone volume and structure. To clarify whether the treatment effects on osteogenic potential observed in isolated BMSCs correlated with changes in density of osteoblasts in bone, cuboidal shaped osteoblasts were enumerated on trabecular bone surface in tibial metaphysis secondary spongiosa (Figure 6e). Consistently, MTX + control IgG treatment reduced the osteoblast density (*p* < 0.05 vs. control), while blocking Notch2 along with MTX treatment attenuated this reduction (*p* < 0.05 vs. MTX + control IgG) (Figure 6f). Furthermore, treatment effects on expression of key osteogenesis regulatory genes were examined. Interestingly, *Runx2* expression was sharply increased in MTX + control IgG treatment group (*p* < 0.01 vs. control) which was attenuated in the MTX + anti-Notch2 group (*p* < 0.01 vs. MTX + control IgG) (Figure 6g). The *Osx* gene expression was upregulated following MTX treatment although it was not statistically significant compared to the control (Figure 6h). However, a significant upregulation in mRNA expression of *OCN* was observed in the MTX + control IgG group (*p* < 0.05 vs. control), which was dampened by Notch2 blockade (*p* < 0.05) (Figure 6i).

### 3.9. Notch2 Blockade in MTX-Treated Rats Was Associated with Changes in Activation of Wnt/β-Catenin Pathway and Expression of Key Wnt Signalling Mediators

**Figure 7:**
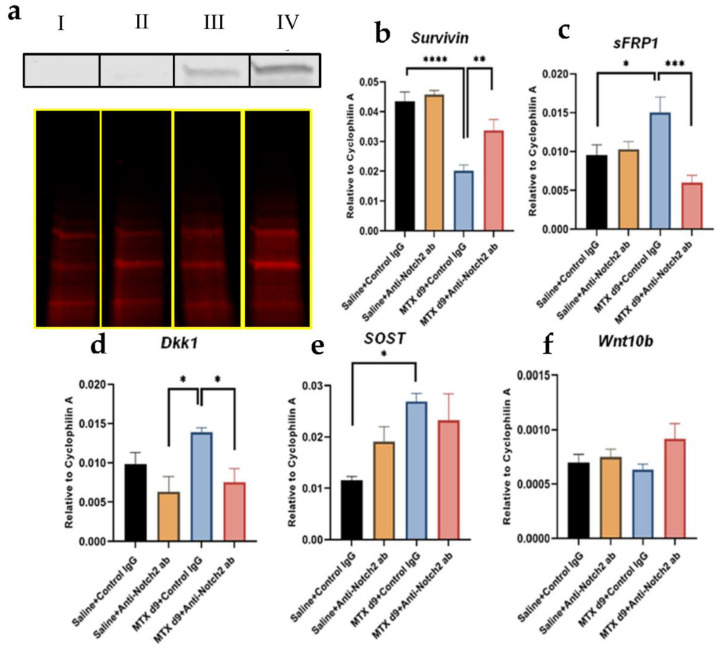
Effects of MTX treatment or MTX with anti-Notch2 antibody treatment on Wnt/β-catenin signalling. (**a**) Representative cytosolic β-catenin protein expression as assessed by Western blot in different groups: (I) control IgG, (II) anti-Notch2 antibody, (III) MTX + anti-Notch2, (IV) MTX + control IgG. Total protein staining has been used as internal loading control. Treatment effects on mRNA expression levels of (**b**) Survivin, (**c**) sFRP1, (**d**) Dkk1, (**e**) SOST, and (**f**) Wnt10b in different groups; * *p* < 0.05, ** *p* < 0.01, *** *p* < 0.001, **** *p* < 0.0001.

Our previous findings suggested that MTX-induced marrow adiposity and bone loss is associated with suppressed activation of the Wnt/β-catenin pathway [15,19]. Since there is evidence supporting crosstalk between Notch and Wnt/β-catenin signalling pathways [37,54], as a means to investigate potential involvement of the Wnt pathway in the Notch2 blockade rescue effects observed above, gene expression of Wnt antagonists, Wnt major ligand and downstream target gene, as well as cytosolic β-catenin protein expression level, was assessed in collected metaphyseal bone samples. After MTX + control IgG treatment, there was a higher protein level of cytosolic β-catenin (an indication of non-activated β-catenin, which will be degraded and will not enter the nucleus to activate the downstream target genes) (Figure 7a and Supplementary Figure S2) and, consistently, a drastically decreased level of mRNA expression of *Survivin* (one Wnt target gene) (*p* < 0.0001 vs. control). However, the blockade of Notch2, together with MTX treatment, significantly attenuated the increase in cytosolic β-catenin level and the decrease in *Survivin* (*p* < 0.01 vs. MTX + control IgG) (Figure 7b). These results suggest that MTX treatment causes a higher cytoplasmic protein level of β-catenin, and that MTX-induced downregulation of β-catenin signalling can be rescued to a good extent by blocking Notch2 signalling.

Next, treatment effects on gene expression levels of the major Wnt antagonists, sFRP1, Dkk1 and SOST, were assessed (Figure 7c–e). Following MTX + control IgG treatment, sFRP1 was significantly upregulated (*p* < 0.05 vs. control), which was attenuated by MTX + anti-Notch2 antibody treatment (*p* < 0.001 vs. MTX + control IgG) (Figure 7c). Similar was true for the induction of Dkk1 expression by MTX + control IgG treatment (*p* < 0.05 vs. control) and the attenuation of this induction with Notch2 blockade (*p* < 0.05 vs. MTX + control IgG) (Figure 7d). Although upregulation of SOST was revealed in MTX + control IgG group (*p* < 0.05 vs. control), it was not statistically significantly altered in MTX + anti-Notch2 antibody treatment group when compared to MTX + control IgG treatment (Figure 7e). Furthermore, assessment of mRNA expression level of Wnt10b (the major Wnt ligand known to regulate bone/fat balance in the bone marrow) revealed no statistically significant changes following treatments (Figure 7f).

## 4. Discussion

With the increased success in childhood cancer treatment with chemotherapy, elevated prevalence of bone-related chronic complications, such as osteoporosis, increased risk of fractures, marrow adiposity and osteonecrosis has been reported, which has reduced the quality of life in the increasing population of cancer survivors [6,7,8,9,10,11]. However, the mechanism for chemotherapy-induced bone damage and subsequent recovery still needs to be elucidated, and currently, there is a lack of specific and safe therapeutic regimens to protect bone from the side effects of chemotherapy. Using a rat model of acute treatment with a most commonly used antimetabolite, MTX, the current study has observed increased activation of the Notch2 pathway following MTX treatment, which is associated with MTX-induced increased osteoclastogenesis and bone resorption, decreased osteogenesis but increased bone marrow adipogenesis. In addition, blockade of Notch2 signalling was found to attenuate these bone pathological changes, which is associated with its effect in ameliorating MTX treatment-induced Wnt/β-catenin signalling defects in the bone.

### 4.1. MTX Chemotherapy Bone Damage Is Associated with Alteration in Notch2 Signalling

Using a rat model of intensive acute MTX treatment, we previously observed the above-mentioned bone pathologies in bones of treated rats, commencing at day 6, peaking at day 9, and recovering at day 14 following the first of the five daily MTX doses [13,14]. As a step to explore potential molecular mechanisms for MTX-induced bone damage, we screened alterations in gene expression of 91 key regulatory factors that are known to regulate bone homeostasis, and we demonstrated that Notch signalling alteration is obvious and that particularly Notch2 receptor over activation is associated with MTX-induced bone loss and marrow adiposity reported previously [15,19]. In the current study, with a neutralising antibody specifically designed against Notch2 negative regulatory region (NRR2) to block Notch2 receptor [44] and used in several studies [19,46], we have illustrated that Notch2 blockade can partially rescue MTX treatment-induced bone/bone marrow damage. Along with the significant role of Notch in bone homeostasis, Notch inhibitor has been clinically evaluated to overcome chemoresistance in some cancers such as ALL and osteosarcoma [55,56], and thus it might be reasonable to speculate targeting Notch signalling along with chemotherapy could serve as a treatment option to protect bone from chemotherapy side effects.

### 4.2. Notch2 over Activation in Bone Is Associated with Imbalanced Bone Turnover Following MTX Chemotherapy

Consistent with findings from our previous studies, a remarkable reduction in trabecular bone volume was observed after MTX treatment. This could be speculated that, apart from direct impact of MTX on healthy bone cells, deregulation in bone turnover might have happened after MTX treatment. In this study we have illustrated that MTX treatment caused increased presence of bone-resorbing osteoclasts on metaphyseal trabecular bone surface and consistently increased osteoclastogenic potential of the isolated non-adherent cells bone marrow cells. Previous observations using the same rat model to study MTX chemotherapy illustrated that MTX treatment induces osteoclastogenesis through increasing mRNA expression levels of RANKL and proinflammatory cytokines (TNF-α, IL-1 and IL-6) in bone [57] and elevating TNF-α protein in serum [16]. Surprisingly, blockade of Notch2 was found to be able to attenuate the increased density of osteoclasts on bone surfaces and to preserve the bone volume and trabecular structure. Consistently, Notch2 blockade was found to diminish MTX adverse effects on osteoclast formation, which is associated with its inhibitory effect on MTX-induced induction in both RANKL and NFATc1. Consistently, previous studies demonstrated that Notch2 activation in osteoclast precursors induces osteoclastogenesis in vitro [58,59], and that Notch2 activation in osteoblasts induces RANKL and enhances osteoclastogenesis [58]. Furthermore, Notch2 over activation has been shown to induce osteoclastogenesis by promoting NFATc1 activity [41]. Thus, our results and previous findings suggest that Notch2 could be a potential therapeutic target to reinstate the bone in this chemotherapy bone loss setting.

In this study, MTX chemotherapy was found to reduce osteoblast density and osteogenesis differentiation of BMSCs in trabecular bone, which could be attenuated with the anti-Notch2 antibody co-treatment. Interestingly, despite our findings of reduced osteogenesis differentiation and osteoblast density, our gene expression analyses revealed that MTX treatment caused upregulation of osteogenic transcription factors (Runx2 and Osx) and osteocalcin (a later stage osteogenesis marker) in the bone. These results were in line with our previous findings of upregulated osteocalcin expression at day 9 following MTX treatment [13,57]. The possible explanation for this unexpected increase in gene expression of osteogenic markers on day 9 (a day with the worst histological damage) might be the compensatory recovery mechanism which has started but it is not enough to allow the bone to recover from damaging effects of MTX. On the other hand, the lack of obvious changes in mRNA expression of these osteogenic markers as a result of Notch2 blockade in MTX-treated rats (compared to control) suggest that administration of anti-Notch2 antibody along with MTX treatment possibly can preserve bone from MTX-induced turnover and therefore maintain bone homeostasis.

### 4.3. Over Activation of Notch2 in Bone Following MTX Treatment Is Associated with Osteogenesis/Adipogenesis Imbalance of BMSCs

The current study revealed MTX-induced reductions in osteoblast density and osteogenic differentiation potential of BMSCs but increases in bone marrow adipocyte density and in BMSC adipogenic differentiation. Given the fact that osteoblasts and adipocytes share common stromal precursors in the bone marrow, our results suggested that MTX treatment changes commitment potential of BMSCs causing a commitment switch in favour of the adipocyte lineage over the osteoblast lineage, which are consistent with previous observations on chemotherapy-induced bone marrow adiposity [18,51]. This change in reciprocal commitment of BMSCs possibly has resulted in reduced bone formation which is not able to compensate bone resorption on day 9 following MTX treatment. Surprisingly, our results revealed that this bone/fat switch was accompanied by increased Notch2 activation in the bone, and that Notch2-neutralising antibody treatment during MTX chemotherapy was able to reduce adipocyte density in marrow cavity and caused a decline in adipogenic potential of BMSCs isolated. While previous findings showed controversial role of Notch receptors and target genes in inhibition or promotion of differentiation of mesenchymal stromal cells to adipocytes [28,60], further studies are required to investigate specific roles of each Notch receptor such as Notch2 in adipogenesis, in both physiologic and pathological conditions.

### 4.4. Alteration in Notch2 Signalling Is Corelated with Changes in Wnt/β-Catenin Pathway Following MTX Treatment

Several pieces of evidence suggest the association between Notch signalling and Wnt pathway in maintaining bone homeostasis. Over activation of Notch signalling through Notch1 upregulation has been illustrated to inhibit osteogenesis by suppressing canonical Wnt pathway [29], and Notch induction in osteoblast lineage cells inhibits their differentiation via suppressing cytosolic β-catenin level [37,38,61]. In contrast, Notch1 overactivation in osteocytes induces Wnt/β-catenin pathway by suppressing Wnt antagonists *Dkk1* and *Sost* [62]. Georgiou et al. have shown that MTX chemotherapy attenuates Wnt/β-catenin pathway at day 6 and 9 after chemotherapy via induction of sFRP1 which is known as a key Wnt antagonist [19]. In the current study, we have observed changes in protein and mRNA level of Notch2 are associated with alterations in β-catenin levels in bone samples. At day 9 following MTX, while there was a higher cytosolic β-catenin level compared to control, β-catenin target gene Survivin was significantly downregulated in this MTX-treated group. Surprisingly, when MTX + anti-Notch2 antibody were given together, Wnt target Survivin was upregulated, and cytosolic β-catenin level was reduced to the control level. Our further investigation revealed that administrating anti-Nocth2 antibody along with MTX treatment alleviates induction of *Dkk1* and *sFRP1*, which suggests Wnt pathway activation can be preserved following the combination treatment. Although more studies are needed to confirm the regulatory role of Notch2 on the Wnt pathway, our results suggest that the partial rescuing role of anti-Notch2 antibody treatment in our MTX rat model could be through its role in regulation of the Wnt pathway.

## 5. Conclusions

**Figure 8:**
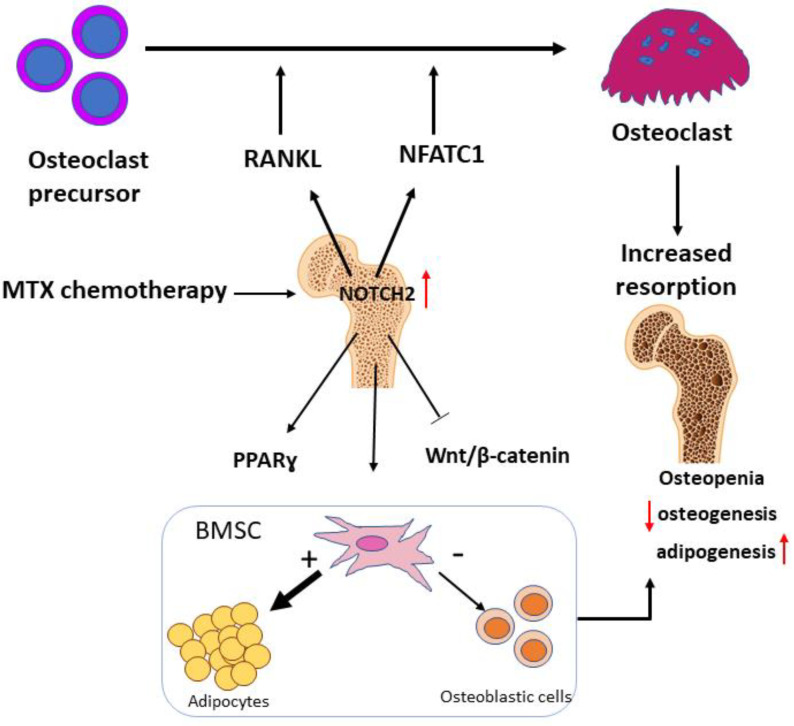
A schematic representation of the roles of the increased Notch2 activation in mediating MTX chemotherapy-induced bone loss and bone marrow adiposity. Increased expression and activation of Notch2 in rat metaphyseal bone following MTX treatment induces expression of receptor activator of NF-κB ligand (RNAKL) and nuclear factor of activated T-cells 1 (NFATc1) that promote osteoclastogenesis and increase the density of osteoclasts on trabecular surfaces, resulting in increased bone resorption. Induction of Notch2 activity following MTX treatment increases the expression of adipogenic transcription factor peroxisome proliferator-activated receptor gamma (PPARγ) and suppresses Wnt/β-catenin signalling in bone which results in decreased osteogenic differentiation potential but increased adipogenic differentiation capacity of bone marrow stromal cells (BMSCs) and consequently reduced osteogenesis and accumulation of fat in marrow cavity: Induction; Inhibition; **+** Increase; **−** Decrease.

Results from our study suggest that over activation of Notch2 signalling pathway plays a key role in MTX treatment-induced bone/bone marrow damage by increasing osteoclastogenesis, adipogenesis and reducing osteogenesis (Figure 8). Targeting Notch2 could be a potential treatment option to overcome MTX skeletal side effects. Considering the roles of Notch2 antagonism in preserving bone homeostasis along with anti-cancer activity of Notch inhibitors [63,64], it is reasonable that targeting Notch2 can be considered as a potential treatment regimen against malignancies and also protecting bone. However, consideration should also be taken into account with potential impact of general administration of Notch2 antagonism on other organs. Our findings may open new revenues for designing new treatments for management of cancer chemotherapy side effects on the skeletal system and for improving the quality of life of cancer patients.

## Figures and Tables

**Table 1:** Primers used in this study.

Primers	Genes	Forward Sequence (5′-3′)	Reverse Sequence (5′-3′)
**Cyclophilin A**	***PPIA***	GAGCTGTTTGCAGACAAAGTTC	CCCTGGCACATGAATCCTG
**Dkk-1**	***DKK1***	GGTTCTTGGTCGTGCTTTCA	CTTGATCGCGTTGGAATTGA
**Nfatc1**	***NFATC1***	GTGCAAGCCAAATTCCCTGG	CTTGGACGGGGCTGGTTATT
**RANKL**	***TNFSF11***	CCGTGCAAAGGGAATTACAAC	GAGCCACGAACCTTCCATCA
**OPG**	***TNFRSF11B***	CACAGCTCGCAAGAGCAAACT	ATATGCCGTTGCACACTGCTT
**Runx2**	***RUNX2***	TCACAAATCCTCCCCAAGTGG	GAATGCGCCCTAAATCACTGA
**Osteocalcin**	***BGLAP***	GCTGGCCCTGACTGCATTCTG	ATTCACCACCTTACTGCCCTCCTG
**Osterix**	***SP7***	GCTTTTCTGTGGCAAGAGGTTC	CTGATGTTTGCTCAAGTGGTCG
**SOST**	***SOST***	CAACCAGACCATGAACCGGG	AAGCGGGTGTAGTGCAGCTC
**PPARγ**	***PPARG***	AACGTGAAGCCCATCGAGGACATC	CTTGGCGAACAGCTGGGAGGAC
**sFRP-1**	***SFRP1***	CCCGAGATGCTCAAATGTGAC	AGATGTTCGATGATGGCCTCC
**β-Catenin**	***CTNB1***	CTTGGCTGAACCGTCACAGAT	TCCTCGTCATTTAGCAGTTTGG
**Survivin**	***BIRC5***	AACTGGCCCTTCCTGGAG	TCAGGCTCGTTCTCGGTAG
**Wnt10b**	***WNT10B***	AGAATGCGGATCCACAACAAC	TCCAACAGGTCTTGAATTGGC
**Notch 1**	***NOTCH1***	CCAGGGTGGTCAGGAAAGTC	GGTTCTGGCTGCACTCGTTA
**Notch2**	***NOTCH2***	ATGCCGGGTTTCAAAGGTGT	ATGTCGATCTGGCACACTGG
**Hes1**	***HES1***	GACACCGGACAAACCAAAGAC	AATGCCGGGAGCTATCTTTCT
**HeyL**	***HEYL***	GTCCCCACTGCCTTTGAGAA	CATCAAAGAACCCTGCGCCA

## Data Availability

The data that support the findings of this study are available on request from the corresponding author.

## Supplementary Materials

The following supporting information can be downloaded at: https://www.mdpi.com/article/10.3390/cells11091521/s1, Supplementary Table S1: PCR array factors essential for bone homeostasis: changes in mRNA expression in metaphyseal bone at day 9 following MTX treatment; Supplementary Figure S1: Metaphyseal bone NICD2 protein expression at different time points as assessed by Western blot with total protein being used as an internal loading control; and Supplementary Figure S2: Cytosolic β-catenin protein expression assessed by Western blot in different groups: (I) control, (II) Anti-Notch2 antibody, (III) MTX+Anti-Notch2, (IV) MTX+control IgG.
