# Supplementary material for: Notch2 Blockade Mitigates Methotrexate Chemotherapy-Induced Bone Loss and Marrow Adiposity
Source: Cells. 2022 May 2;11(9):1521. doi: 10.3390/cells11091521 (PMC9103078; doi:10.3390/cells11091521)
Supplement: Supplementary file 1 [file cells-11-01521-s001.zip › cells-1667388-supplementary.pdf]

Supplementary Materials:

Supplementary Table S1. PCR array factors essential for bone homeostasis: changes in mRNA expression in metaphyseal bone at day 9 following MTX treatment.

Target	Log_2 Fold Change	Target	Log_2 Fold Change	Target	Log_2 Fold Change	Target	Log_2 Fold Change
Ifna1	0.50	Pgf	-0.70	Nrp2	0.14	Tymp	-1.11
Thbs1	0.85	F3	1.40	Cxcl1	-0.24	Mapk14	0.19
Ang	1.95	Il1b	0.76	Col18a1	-1.59	Fgf1	1.39
Eng	-2.39	Nrp1	0.20	Serpinf1	0.04	Pdgfb	-1.72
Igf1	0.06	Ctgf	0.02	Itgb3	0.29	Fgf6	0.28
Vegfc	-1.03	Sphk1	-2.05	Tgfb2	0.48	Angpt2	1.01
Plau	1.69	Efna1	0.03	Plg	2.10	Vegfb	-2.07
Vegfa	-1.19	Hif1a	-0.03	Tgfb1	-1.19	Pdgfa	-1.84
Egf	1.84	Il6	0.44	Ptgs1	0.54	Itga5	1.06
Mmp2	-0.31	Ccl2	0.47	Fgfr3	-2.20	Col4a3	-0.59
Tgfb3	0.95	Tnf	-1.88	Serpinb5	0.29	S1pr1	0.13
Hgf	0.43	Tgfa	0.87	Fgf2	0.98	Timp3	1.22
Edn1	0.70	Flt1	1.44	Mmp3	0.60	Angpt1	0.50
Slit3	-0.14	Figf	0.60	Serpine1	0.78	Jag2	-3.13
Ptk2	0.56	Mdk	-2.40	Mmp19	0.37	Ifnb1	0.41
Cdh5	0.24	Itgav	0.01	Tgfbr1	-0.37	Fgf23	1.48
Lect1	1.27	Mmp14	-0.20	Lep	2.09	Dll1	0.37
Mmp9	-0.66	Akt1	-2.22	Kdr	0.47	Fgf9	0.52
Cxcl9	1.85	Tie1	0.17	F2	0.45	Tnfsf11	0.71
Timp2	0.61	Tek	0.10	Epas1	-0.26	Bmp2	-2.16
Dll3	-0.65	Cxcl2	0.60	Nos3	-1.61	Bmp4	-1.37
Fn1	-0.40	Ifng	0.16	Jag1	0.21	Bmp7	-1.69
Id1	-0.22	Timp1	-0.22	Erbb2	-1.18	-	-

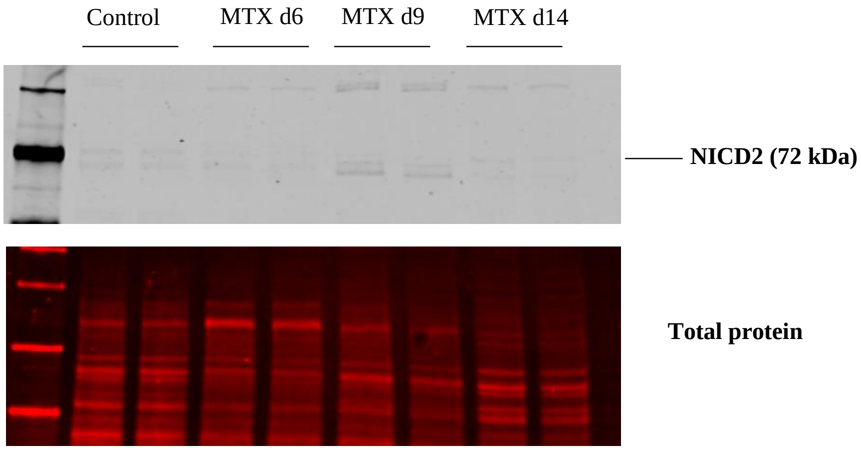

Supplementary Figure S1. Metaphyseal bone NICD2 protein expression at different time points as assessed by Western blot with total protein being used as an internal loading control.

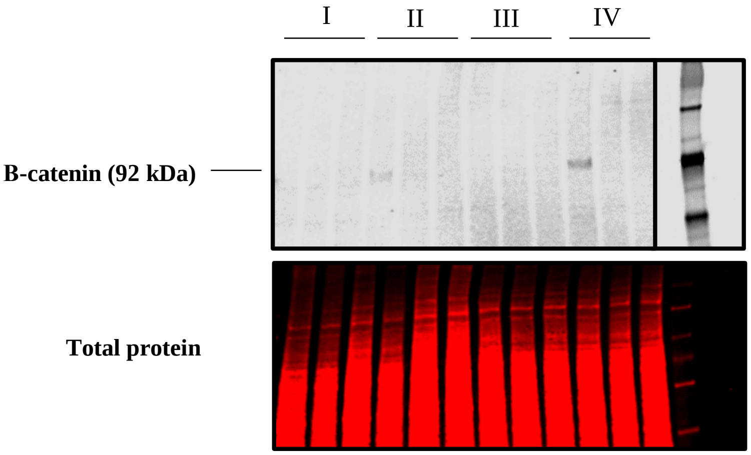

Supplementary Figure S2. Cytosolic β-catenin protein expression assessed by Western blot in different groups: (I) control, (II) Anti-Notch2 antibody, (III) MTX+Anti-Notch2, (IV) MTX+control IgG.
